# Approaches for Identifying LncRNA-Associated Proteins for Therapeutic Targets and Cancer Biomarker Discovery

**DOI:** 10.3390/targets3030027

**Published:** 2025-08-11

**Authors:** Mohammad Shabir Hussain, Puneet Vij, Sudhir Kotnala, Shadab Ahmad, Subhash C. Chauhan, Manish K. Tripathi

**Affiliations:** 1 Medicine and Oncology ISU, School of Medicine, The University of Texas Rio Grande Valley, McAllen, TX 78504, USA;; 2 South Texas Center of Excellence in Cancer Research, School of Medicine, The University of Texas Rio Grande Valley, McAllen, TX 78504, USA; 3 Department of Pharmaceutical Sciences, St. John’s University, Queens, NY 11439, USA;; 4 Centre for Cellular and Molecular Platforms (C-CAMP), GKVK Post, Bellary Rd, Bengaluru 560065, Karnataka, India;

**Keywords:** long non-coding RNAs (LncRNAs), proteomics, LC–mass spectrometry, cancer biomarkers, therapeutic targets, protein interactions

## Abstract

Long non-coding RNAs (lncRNAs) are increasingly recognized as key regulators of gene expression and cellular signaling in cancer. Their functions are primarily mediated through interactions with specific protein partners that modulate chromatin structure, epigenetic remodeling, transcription, and signal transduction. In this review, we explore reports and strategies for the proteomic characterization of lncRNA-associated proteins, particularly emphasizing high-throughput liquid chromatography–mass spectrometry (LC-MS)-based techniques. Affinity-based methods such as RNA pull-down, ChIRP MS, RAP-MS, BioID-MS, and SILAC-MS enable sensitive and specific mapping of lncRNA and protein complexes. These approaches reveal cancer-specific proteomic signatures, post-translational modifications, and mechanistic insights into tumor biology. The use of label-free quantification, bituminization, and crosslinking strategies further enhances the resolution of dynamic RNA–protein networks. Validation tools following bioinformatic analyses, such as Western blotting, immunohistochemistry, immunofluorescence, and ELISA, are used to prioritize and confirm findings. Candidate biomarkers from hepatocellular carcinoma to colorectal and prostate cancers, profiling lncRNA-associated proteins, hold promise for identifying clinically actionable biomarkers and therapeutic targets. This review highlights the translational relevance of lncRNA protein studies and advocates for their broader adoption in oncological research. In LC-MS workflows, proteins bound to lncRNAs are enzymatically digested into peptides, separated via nano-LC, and analyzed using high-resolution tandem MS. Label-free or isotope-labeled methods quantify differential enrichment, followed by bioinformatics-driven pathway annotation.

## 1. Introduction

Long non-coding RNAs (lncRNAs) represent a diverse and functionally significant class of RNA molecules that do not encode proteins but play critical roles in regulating gene expression and cellular functions [1]. The isolation, identification, and quantification of proteins associated with lncRNAs are challenging to comprehend [1–3]. Unlike proteins, which can often be studied using antibody-based techniques, lncRNAs require different investigative methods due to their distinct characteristics and interactions. LncRNAs can act as scaffolds, combining various molecular components to form functional complexes essential for chromatin remodeling, transcription regulation, and RNA processing [4]. Their unique regulatory roles and involvement in diverse biological processes underscore the need for specialized techniques to study these molecules comprehensively, beginning with their identification and characterization. High-throughput sequencing technologies, such as RNA-seq, have been instrumental in identifying lncRNAs across different tissues and disease conditions, providing a comprehensive view of the transcriptome and revealing novel lncRNAs along with their expression patterns [5]. Bioinformatics tools predict the secondary structures of lncRNAs and identify potential binding sites for proteins, RNA, and DNA, while experimental techniques like RNA immunoprecipitation and crosslinking immunoprecipitation validate these interactions in vivo [6]. One such innovative approach involves isolating lncRNAs using complementary oligonucleotide probes that act as primers. These probes hybridize specifically to the lncRNA of interest, allowing for its selective enrichment from a complex RNA mixture [7]. Once isolated, the lncRNA can be tagged with biotin, facilitating subsequent purification steps and enhancing the detection and analysis of the lncRNA and its associated proteins or other interacting molecules [8]. A second primer, complementary to a different region of the lncRNA, can further amplify or label the RNA, providing a particular and efficient way to study lncRNA interactions within the cell [9]. Advanced techniques are particularly valuable in cancer research, where dysregulated lncRNAs play key roles in regulating genes involved in cell proliferation, apoptosis, and metastasis. These lncRNAs often interact with proteins that serve as hallmarks of cancer, including those associated with oncogenic signaling pathways and tumor suppressor functions. For example, the lncRNA *MALAT1* has been shown to interact with specific proteins that promote lung cancer metastasis [10]. Understanding these interactions is crucial for developing targeted therapies. By isolating and studying lncRNAs, i.e., *HOTAIR* [11], researchers can identify chromatin-modifying complexes involved in cancer and design drugs that can disrupt these interactions to inhibit cancer-promoting effects [12]. The therapeutic potential of lncRNAs extends beyond cancer. LncRNAs are involved in various diseases, including cardiovascular diseases, neurological disorders, and metabolic diseases. For example, the lncRNA *ANRIL* is implicated in atherosclerosis and cardiovascular risk, while *NEAT1* is associated with neurodegenerative diseases [1,13]. Targeting these specific lncRNAs could provide a novel therapeutic strategy for these conditions. Moreover, the specificity and distinct expression patterns of lncRNAs make them attractive candidates for non-invasive cancer biomarkers [14,15]. For example, the lncRNA *PCA3*, specific to prostate cancer, can be detected in urine, providing a non-invasive diagnostic tool [16–19]. High-throughput sequencing technologies and bioinformatics tools have advanced our understanding of lncRNAs’ regulatory roles and interactions. Techniques like RIP, CLIP, and CRISPR/Cas9-mediated genome editing have enabled researchers to comprehensively map the interactome of lncRNAs and study their molecular functions [20]. These methods have revealed the essential roles of lncRNAs in development, differentiation, and various diseases. *MALAT1* interacts with splicing regulators such as RBFOX2 and promotes epithelial-to-mesenchymal transition in ovarian and lung cancers, highlighting a targetable vulnerability in metastatic progression. These technologies follow our discussion of dysregulated lncRNA functions, enabling a more logical transition into experimental methodologies.

In short, lncRNAs are critical regulators of gene expression and cellular functions, with unique roles distinguishing them from protein-coding genes. Advanced techniques for studying lncRNAs, such as oligonucleotide probes, biotin tagging, RIP, CLIP, and CRISPR/Cas9-mediated genome editing, have significantly enhanced our understanding of their biology. These techniques are particularly valuable in cancer research, where lncRNAs play crucial roles in oncogenesis and tumor progression. Identifying lncRNA-associated proteins and understanding their interactions can reveal novel therapeutic targets and biomarkers, which can help pave the way for innovative treatments for cancer and other diseases. The identified differentially expressed or modulated proteins can be validated using commercially available antibodies through Western blotting and immunohistochemistry (IHC), enzyme-linked immunosorbent assays (ELISAs), and immunofluorescence (IF). These identified proteins can also be validated using different patient cohorts worldwide.

This approach can uncover novel molecular signatures that could be useful in the diagnosis and prognosis of cancer and other associated diseases, such as chronic liver disease (CLD), acute-on-chronic liver failure (ACLF), nonalcoholic fatty liver disease (NAFLD), severe alcoholic hepatitis (SAH), and other human diseases [2–5]. This review highlights key advances and strategies in the proteomic profiling of lncRNA-associated proteins, focusing on high-throughput LC-MS-based approaches. Affinity-based techniques, including RNA pull-down, ChIRP-MS, RAP-MS, BioID-MS, and SILAC-MS, offer powerful tools for the sensitive and specific identification of lncRNA–protein interactions. Quantitative analysis of differentially enriched proteins is performed using either label-free or isotope-labeling LC-MS approaches, and the resulting data are subjected to bioinformatics-driven pathway analysis and functional annotation. The proteins associated with lncRNAs are first trypsin digested into peptides, which are then separated by high-resolution MS.

A study by [6] focused on Metastasis-Associated Lung Adenocarcinoma Transcript 1 (*MALAT-1*) and its potential role as a diagnostic or prognostic biomarker for ovarian cancer. MALAT-1 expression was found to be significantly upregulated in ovarian cancer tissues compared to normal tissues. The findings suggest that *MALAT-1* could serve as a therapeutic target due to its involvement in cancer progression, highlighting its importance in tumor growth and metastasis. Another explored Urothelial Cancer-Associated 1 (*UCA1*) in gastric cancer, demonstrating that *UCA1* interacts with miR-145 and MYO6, forming a regulatory axis that affects cancer cell proliferation and apoptosis, suggesting new therapeutic approaches targeting regulatory axis [7]. Others examined the impact of metformin on cancer risk in type 2 diabetes patients, suggesting that metformin might reduce cancer risk and highlighting its potential as a chemo-preventive agent. Another study utilized techniques such as RAPID-SELEX and RNA-competitive to map lncRNA–protein interactions, providing insights into lncRNA cellular mechanisms and their disease-related perturbations, and further used MS2 trapping and SILAC-based display to identify lncRNA-bound proteomes, aiding in understanding lncRNA functions in cellular networks [8]. Another study focused on Highly Upregulated in Liver Cancer (*HULC*), demonstrating its overexpression in various cancers and promoting tumor growth, suggesting its potential as a biomarker and therapeutic target [9]. It was shown that *HULC* interacts with *LDHA*, promoting glycolysis in cancer cells and highlighting *HULC’s* role in cancer metabolism [10]. Other work identified nearly 8000 cancer-specific lncRNAs, including *PCA3* for prostate cancer, emphasizing their diagnostic and prognostic potential. A study suggested that M1 exosomes and *HOXA* Transcript at the Distal Tip (*HOTTIP*) polarize monocytes into an antitumor phenotype, suggesting a novel approach for immunotherapy [11] demonstrated that *HOTAIR* mediates gene silencing and enhances tumor progression, suggesting its use alongside existing therapies to sensitize tumors [12]. Another study showed that *CCAT1* regulates miR-490-3p in ovarian cancer, indicating new therapeutic strategies [13], and then explored *CCAT2*′*s* co-expressed genes, suggesting targeting *CCAT2* pathways for cancer therapy. *H19* was found to enhance cancer cell proliferation and glycolysis by downregulating miRNA-519d-3p and upregulating *LDHA*, highlighting its potential as a therapeutic target. It has been demonstrated that *CCAT1-L* inhibits *EMT* in gastric adenocarcinoma cells, suggesting its potential to prevent cancer metastasis. Others identified *CRNDE* as a marker and therapeutic target against chemoresistance in gastric cancer [14] and showed that *FER1L4* regulates neural stem cell proliferation and differentiation, suggesting its therapeutic potential in neurodevelopmental disorders. Finally, it was demonstrated that *PTENP* inhibits cell proliferation and *EMT* while inducing apoptosis in cervical cancer cells, highlighting its potential as a therapeutic target as well [15]. These brief explanations of various studies are examples of LncRNA-associated proteins and their further use in liver, colorectal, and several other different types of cancers.

## 2. Approaches for Identifying Protein Interactions with LncRNAs

The identification of proteins interacting with lncRNAs is fundamental to elucidating the molecular mechanisms governing their functional roles and regulatory pathways. One such approach, the RNA Antisense Purification coupled with Mass Spectroscopy (RAP-MS) technique, enables precise identification of direct RNA–protein interactions in vivo (within living cells), significantly advancing our capacity to map RNA-centered regulatory networks. Employing whole cell lysates or nuclear extracts, the RAP-MS workflow yields a comprehensive profile of proteins intricately associated with the target lncRNA within physiologically relevant cellular contexts. A critical component of this method involves the rational design of suitable-length antisense oligonucleotides tiled across the lncRNA sequence, with stringent optimization to minimize off-target hybridization, validated through LC-MS data analysis. Additionally, 5′-biotin modification to the antisense oligonucleotide enables efficient affinity purification of lncRNA–protein complexes. Studies targeting lncRNA-associated protein assemblies in HeLa cells further highlight the robustness and broad applicability of this technique across diverse experimental systems. Furthermore, advancements such as the HyPR-MS method have enhanced efficiency and scope of RNA–protein interactome analysis. Oligonucleotide-based affinity purification approaches continue to empower the systematic isolation of lncRNA–protein complexes from complex biological matrices [16,17]. In HeLa cells, such methods have robustly delineated interaction networks; notably, *MALAT1* engages with the splicing factor *SRSF1*, while *NEAT1* associates with the paraspeckle proteins *NONO* and *PSPC1* [18]. A significant innovation in this realm, the HyPR MS technique of hybridization and purification of RNA and protein complexes, followed by nano-LC–mass spectrometry analysis has enabled simultaneous, multiplexed purification of distinct RNAs (e.g., *MALAT1, NEAT1*, and *NORAD*), leading to sensitive, high-throughput characterization of their protein interactions in a single experiment or targeted protein identification [19]. To expand beyond RNA and protein complexes, RNA Antisense Purification RNA sequencing (RAP RNA) was introduced to facilitate transcriptome-wide mapping of RNA–RNA interactions via targeted oligonucleotide pull-down followed by high-throughput sequencing [20]. This method revealed both direct and associated protein-mediated RNA duplexes, as exemplified by U1 snRNA binding to 5′ splice-site motifs and the indirect engagement of *MALAT1* with nascent transcripts [21]. Together, these advanced methodologies, HyPR MS and RAP RNA, constitute a powerful toolkit for elucidating the molecular interconnections of lncRNAs, spanning both their proteome and genome (interactome and RNA) [22].

lncRNAs frequently exert their biological functions by forming ribonucleoprotein complexes with specific proteins. For example, the lncRNA HOTAIR interacts with the Polycomb Repressive Complex 2 (PRC2), including the protein EZH2, to facilitate H3K27 trimethylation and transcriptional repression at target genes [23,24]. Another example is NEAT1, which serves as a structural scaffold for paraspeckle nuclear bodies, assembling proteins such as NONO, SFPQ, and PSPC1 that regulate gene expression under stress conditions [25]. Additionally, MALAT1 forms complexes with serine/arginine-rich splicing factors (e.g., SRSF1), modulating alternative splicing [26,27]. These examples illustrate that lncRNA–protein complexes are not only prevalent but also essential for the regulatory capacity of lncRNAs in diverse cellular processes. Therefore, studying these complexes provides critical insight into the mechanistic basis of lncRNA function. These examples reinforce the idea that while not all lncRNAs may rely on protein interactions, the formation of functional lncRNA–protein complexes is a widely observed and biologically significant mechanism. This technique, exemplified in the study of XIST lncRNA localization during X-chromosome inactivation, offers invaluable insights into lncRNA-mediated chromatin regulation. Complementary methods, such as Chromatin Isolation by RNA Purification (ChIRP) and Capture Hybridization Analysis of RNA Targets (CHART), provide high-throughput avenues for elucidating RNA-bound proteins and genomic binding sites of specific lncRNAs. Using a pool of short complementary DNA oligonucleotide probes inspired by *RNA FISH, CHART* adapts an RNase H mapping assay, offering nuanced approaches tailored to different experimental contexts. Additionally, Reversible Crosslinked Immunoprecipitation (ReCLIP) emerges as a powerful tool, keeping intact the loose protein associations that identify lncRNA-associated proteins. By leveraging cell-permeable, thiol-cleavable crosslinkers and in-cell crosslinking, ReCLIP captures endogenous protein–protein interactions with remarkable fidelity, offering a glimpse into the dynamic landscape of RNA–protein interactions within living cells. Interaction with protein complexes is a common mechanism by which lncRNAs exert their functions. Thus, identifying proteins associated with lncRNAs is critical for understanding the molecular mechanisms and functions of lncRNAs. Immunoprecipitation is commonly used to isolate protein complexes associated with a protein of interest. However, this method is not applicable to lncRNAs because antibodies do not recognize RNA. Proteins with higher MS counts in cells transfected with experimental lncRNA plasmids versus empty plasmids are selected as potential candidates. Knockdown of these protein candidates with short hairpin RNAs (shRNAs) is used to confirm the functionality of the lncRNA of interest. Further validation of the binding of protein candidates to the lncRNA of interest is required to verify their association [1,2].

### 2.1. Pathways Associated with LncRNAs

LncRNA-associated proteins are emerging as significant players in diagnosing and treating various cancers [27]. The extensive discovery and reporting of lncRNA-associated proteins highlight their diverse expression patterns and tumor specificity across different cancer types [28–30]. Table 1 lists several key lncRNAs, such as *MALAT1, UCA1, HULC, HOTTIP, CCAT1, CCAT2,* and *H19*, and their identified associated proteins, mentioning methods used for their isolation and identification. These proteins serve as potential biomarkers for the treatment of liver and colorectal cancer patients [31–34]. Additionally, one notable example is the long non-coding RNA Highly Upregulated in Liver Cancer (*HULC*). These proteins may also contribute to mechanistic and pathway analyses of lncRNAs, thereby advancing our understanding of cancer pathophysiology.

### 2.2. Proteomic Approaches for Characterization of LncRNA–Protein Interactions

**Figure 1.**
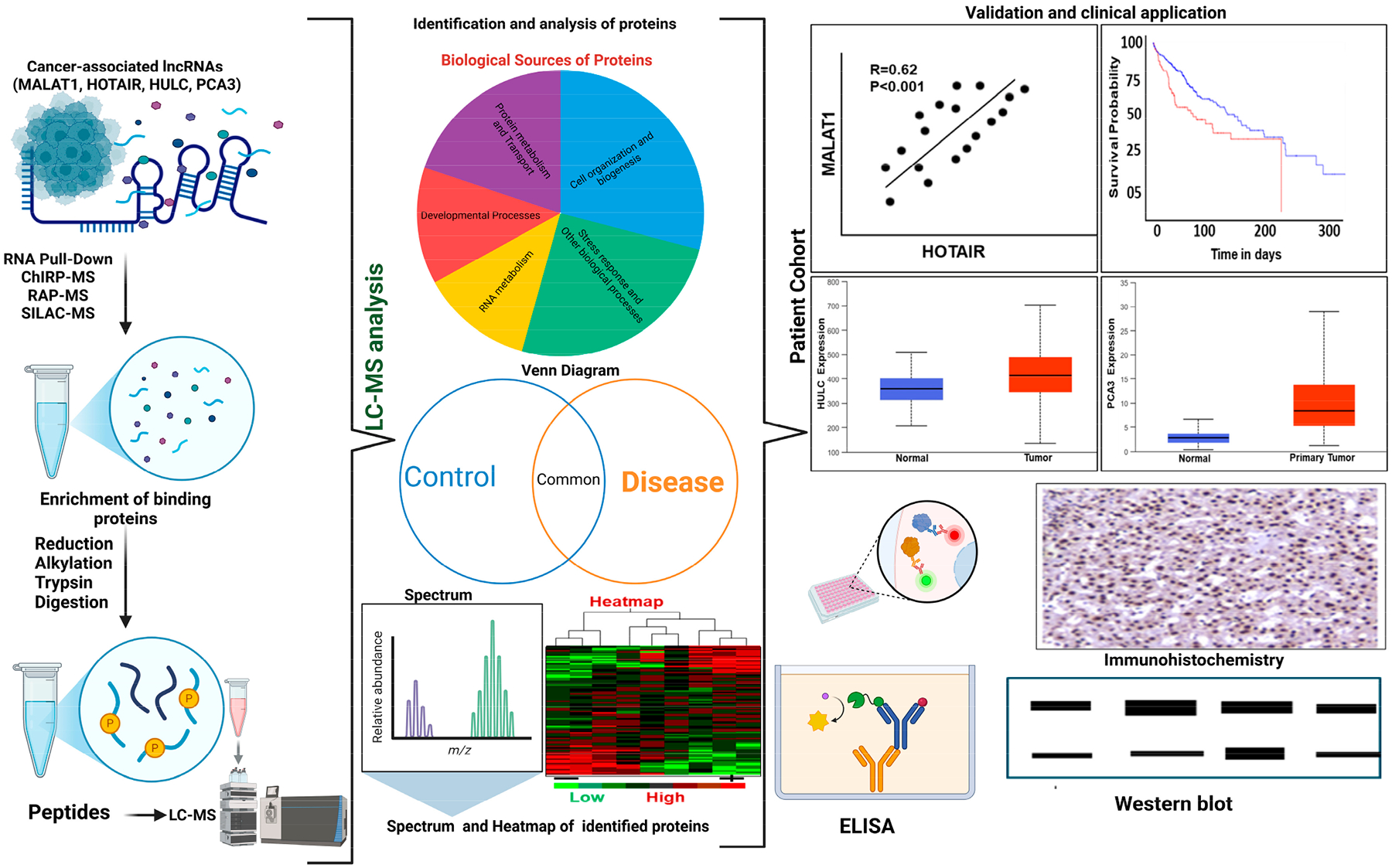
Schematic of the workflow for identifying, analyzing, and validating cancer-associated lncRNA-binding proteins for lncRNAs such as *MALAT1, HOTAIR, HULC*, and *PCA3* using proteomic strategies. Left panel: Cancer-associated lncRNAs involved in various biological processes are subjected to RNA pull-down, using CHIRP-MS, RAP-MS, or SILAC-MS methods to enrich binding proteins. Following enrichment, proteins are reduced, alkylated, and digested, resulting in peptides.

Identifying long non-coding RNA (lncRNA)-associated proteins utilizing proteomics involves diverse methodologies. The assays, like RNA pull-down in lung, gastric, and colorectal cancers, involve BC-IMPAD, NEAT1/miR-17-5p/TGFβR2, and FENDRR-GSTP1, respectively [51]. ChIRP-MS identified *MaTAR25-*Tensin1, *m6A-TP53TG1-CIP2A*, and *CRLM1-hnRNPK* in breast, gastric, and liver cancers, respectively [52–54]. RAP-MS in liver cancer revealed *lincNMR-YBX1/RRM2* interactions driving cancer mechanisms. Moreover, HyPR-MS maps prostate cancer complexes (*MALAT1/NEAT1/NORAD*) [55], while TOBAP-MS identifies HULC’s 140 interactors in liver cancer [56], and BioID-MS links *HOTAIR* to ribosomes in breast cancer cell lines. SILAC-MS quantifies lincNMR and boosts tumor proliferation through a *YBX1-RRM2-TYMS-TK1* axis in liver, lung, and breast cancer cell lines, while *GLCC1* mediates metabolic reprogramming in colorectal cancer and *HULC* (Highly Upregulated in Liver Cancer) [34,56]. Utilizing innovative proteomics methods has become critical for determining exactly how lncRNAs interact with proteins, especially in cancer research. RNA pull-down assays have made it possible to understand the lncRNA–protein networks present in different malignancies, for instance, *BC-IMPAD* in lung cancer, *NEAT1/miR-17-5p/TGF*_*β*_*R2* in gastric cancer, and *FENDRR-GSTP1* in colorectal cancer [56]. Procedures such as ChIRP-MS reveal remarkable collaboration, such as *MaTAR25-*Tensin1 in breast cancer, m6A-modified *TP53TG1-CIP2A* in gastric cancer, and CRLM1-hnRNPK in liver cancer [57,58]. Assays such as RAP-MS have revealed the role of lincNMR and its interaction with *YBX1* and *RRM2*, leading to liver cancer. HyPR-MS has been instrumental in locating prostate cancer-related complexes, including MALAT1, *NEAT1*, and *NORAD*. Additionally, regarding TOBAP-MS, researchers have identified 140 proteins interacting with HULC, showing its significance in liver cancer. BioID-MS networked *HOTAIR* to ribosomes in breast cancer cells, uncovering a further dimension of complexity. Moreover, SILAC-MS has demonstrated how lincNMR accelerates tumor growth through the *YBX1-RRM2-TYMS-TK1* axis in liver, lung, and breast cancer cells [59,60]. Finally, researchers have observed the role of *GLCC1* in metabolic modifications in colorectal cancer, emphasizing the diverse approaches by which lncRNAs influence cancer mechanisms. Table 2 decodes the roles for lncRNAs in linking mechanistic understandings to clinical translation. Significant differences involve the requirement of specific RNA amounts; for instance, ChIRP-MS can work with low-abundance RNA, while RNA pull-down requires high levels. Other assays are based on output specificity, such as HyPR-MS, which allows multiplexed networks (*MALAT1/NEAT1*); BioID-MS, which captures transient interactions; and SILAC-MS, which quantifies shifts, while TOBAP-MS determines stable complexes (*HULC*) [34] and is highly upregulated in liver cancer. These assays demonstrate lncRNA’s role in tumor progression, balancing interaction stability, scope, and biological relevance. Figure 1 illustrates a multi-step pipeline used to identify and validate lncRNA-binding proteins with potential clinical applications.

Peptides are analyzed by LC-MS, LFQ, or TMT label analysis methods. The lncRNA-associated proteins are subjected to sample preparation before injection of the samples, and the isolated peptides are separated by flow of nano-LC (C18 column), analyzed using high-resolution MS (HR-MS/MS). Middle panel: The proteomic data are analyzed to identify the set of proteins associated with the lncRNA isolated during the pull-down. For the biological sources of identified proteins, a Venn diagram shows the total proteins, in controls and experiments, along with the overlap. Heat maps give a differential profile of the identified proteins, which can be further grouped in different biological pathways and spectra of the identified proteins. Right panel: Candidate cancer-associated lncRNAs proteins are validated using multiple approaches, including analysis of patient cohort data (gene expression, survival analysis, etc.), immunohistochemistry (IHC), immunofluorescence (IF), and Western blotting. These results help establish the roles of the identified lncRNA proteins as potential diagnostic or prognostic novel biomarkers and therapeutic targets.

## 3. Discussion

Cancer is the second leading cause of death worldwide and is often characterized by high aggressiveness and resistance to treatment. Several associated lncRNAs have been identified as key regulators of molecular pathways linking to cancer pathogenesis, particularly those involved in inflammation and tumor development. Although their specific roles remain to be fully elucidated, many are taught to participate in post-transcriptional deregulation and are being widely studied as candidate biomarkers for diagnosis, prognosis, and therapy for all kinds of cancers [68–70]. In this article, we highlight emerging lncRNA-associated proteins that play significant roles in cancer progression and may offer potential in predicting treatment outcomes and informing therapeutic strategies [29,71]. For instance, the lncRNA *Malat1* has been proposed as a predictive biomarker for lung cancer metastasis [72]. Beyond lung cancer, Malat1 has also been implicated in breast gynecological and gastrointestinal cancer. Its association with poor overall survival (OS) relapse free survival (RFS), and disease-free survival (DFS) across multiple cancer types further supports its utility as a prognostic marker [72,73]. Malat1 is located on chromosome 11q13 and modulates several tumorigenic signaling pathways, including *MAPK/Erk, PI3Akt, beta catenin/Wnt, Hippo, VEGF*, and *YAP* pathways [72,74]. Additionally, *Malat1* has also been shown to promote ovarian cancer through the regulation of splicing factor *RBFOX2* [73,75]. There are several types of lncRNAs, which we mention in Tables 1 and 2, that play crucial roles in regulating the initiation and progression of various types of cancers [76]. Recent studies have demonstrated that lncRNA-associated proteins can influence chromatin remodeling and epigenetic regulation, contributing to cancer cell plasticity and heterogeneity [77]. For example, the interaction between *HOTAIR* and the *PRC2* complex is known to promote metastasis by silencing tumor suppressor genes [78–80]. Emerging evidence also suggests that lncRNAs may modulate immune cell infiltration and the tumor microenvironment, thus affecting response to immunotherapy [81]. Advanced proteogenomic approaches are now being employed to map lncRNA–protein interaction networks, providing novel insights into cancer biology and treatment resistance mechanisms [82]. A variety of other lncRNAs with critical roles in the initiation and progression of different cancers are summarized in Table 1. This review focuses on the identification of lncRNA-associated proteins, their functional and regulatory roles, and their potential as diagnostic, prognostic, and therapeutic targets in diverse cancer types [76]. Furthermore, we explore proteomic strategies for discovering new biomarkers and therapeutic targets in oncology.

## 4. Conclusions and Future Perspectives

In recent years, long non-coding RNAs (lncRNAs) have emerged as important players in cancer biology, not just because of what they do alone but because of the proteins they interact with. These lncRNA-associated protein partnerships influence everything from gene expression to cell signaling, and in many cases, they help drive cancer progression. This review pulled together current knowledge on how proteomics, especially LC-mass spectrometry-based techniques/method, can help identify these interactions in detail. Tools like RNA pull-down, CHIRP-MS, RAP-MS, and newer methods like HyPR-MS and BioID-MS are giving researchers a clearer view of how lncRNAs function within real biological systems with cancer biology. Still, working with lncRNAs is not easy. Many of them are expressed at low levels, and their interactions can be weak or fleeting, making it tough to detect the right partners with confidence. That is why validation using methods like Western blotting, immunoprecipitation, or patient data is just as important as discovery. Applying the bioinformatics approach for pathway correlation to proteomes using transcriptome analysis and lncRNA-bound proteins requires trypsin digestion for the preparation of peptides and separated by high-resolution MS.

On the clinical side, it is exciting to see that several well-studied lncRNAs like *MALAT1, HULC, HOTAIR*, and *PCA3* are not only linked to cancer progression but also show potential as biomarkers or even therapeutic targets.

However, turning these findings into actual drugs or diagnostic tests is still a work in progress. The field needs better tools, deeper mechanistic insights, and a stronger focus on how these lncRNA-associated protein interactions actually affect patient outcomes. Looking ahead, combining proteomics with other ‘omics’ technologies like transcriptomics and epigenomics could provide a more complete picture of how lncRNAs operate in different cancers. More standardized protocols and computational tools will also help make studies more reproducible and clinically meaningful. In short, the study of lncRNA-associated proteins is a growing and promising area. With continued innovation and collaboration across disciplines, it has the potential to unlock new strategies for diagnosing and treating cancer in a more precise and personalized way.

## Figures and Tables

**Table 1.** Identified lncRNA-associated proteins and utilized methods.

S No.	LncRNAs	Specific Method	Remarks/Conclusions	References
1	*MALAT-1*	Patient samples and ovarian cancer cell lines (SKOV3 and CAOV3)	*MALAT-1* is a diagnostic or prognostic biomarker or therapeutic target for many cancers.	[35]
2	*UCA1*	Via the miR-145/MYO6 axis	The *UCA1*/miR-145/MYO6 axis may serve as a potential therapeutic target for gastric cancer.	[36]
3	*T2D/HCC*	NAFLD/T2D-associated HCC	Metformin may reduce the risk of cancer in patients with T2D. The unadjusted odds ratio was 0.86 (95% CI 0.73 to 1.02). The unadjusted odds ratio for any exposure to metformin since 1993 was 0.79 (0.67 to 0.93 lncRNA–protein interactions in the context of T2D or HCC).	[4,37]
4	Revealing protein	RAPID-SELEX, RNAcompete, RNA Bind-n-Seq, and RNA-Ma	Better understanding of lncRNA cellular mechanisms and their disease-associated perturbations.	[37,38]
5	LncRNA interaction	MS2 trapping, SILAC-based phage display, and protein arrays	LncRNA-bound proteome, or if still-uncharacterized protein domains and architectures are involved, the network will be high.	[37]
6	*HULC*	Tumorigenesis test in vitro and in vivo: RT-PCR and W. B.	Potential implications in cancer diagnosis and therapy.	[33,37]
7	*HULC*	*HULC* interacts with the glycolytic enzyme LDHA	*HULC* promotes the Warburg effect by orchestrating the enzymatic activities of glycolytic enzymes.	[34,39]
8	*Linc00152*	Human tissue samples	Targeting *YAP1/LINC00152/FSCN1* signaling axis prevents the progression of colorectal cancer.	[40]
9	*HEIH*	Non-coding RNAs	Nearly 8000 cancer-specific lncRNAs have been nominated; *PCA3* is a prostate-specific prognostic biomarker for prostate cancer.	[41]
10	*HOTTIP*	In silico analysis, plasmid construction and transfection	Significantly, M1 exosomes and *HOTTIP* polarize circulating monocytes into the antitumor M1 phenotype, which may provide novel insight into HNSCC immunotherapy.	[11]
11	*HOTAIR*	*HOTAIR*-mediated gene silencing	It could be used in conjunction with current drugs to sensitize tumors to the existing therapies	[12]
12	*CCAT1*	RT-qPCR to level of miR-490-3p and *CCAT1*	Facilitate the development of novel therapeutic therapies for treating ovarian cancer.	[42]
13	*CCAT2*	*BOP1-AURKB* signaling	Overexpression of *CCAT2* in colon cells promotes CIN and carcinogenesis by stabilizing and inducing expression of BOP1, an activator of aurora kinase B.	[43,44]
14	*H19*	Enhancing the growth and cell cycle of cancer by EMT induction	Increased proliferation, glycolysis induction, and miRNA-519d-3p downregulation by H19 to increase LDHA expression.	[45]
15	CCAT1-L	Quantitative real-time PCR and Western blotting, respectively	Inhibits epithelial-mesenchymal transition of gastric adenocarcinoma cells and thus suppresses the gastric adenocarcinoma metastasis.	[46]
16	*CRNDE*	Chemosensitivity of GC in clinical samples and a PDX model	Highlights the significance of CRNDE as a potential prognostic marker and therapeutic target against chemoresistance in GC.	[47]
17	*FER1L4*	The cells were extracted from the embryos of rats	FER1L4 modulates the proliferation and differentiation of NSCs via regulating Ascl2.	[48]
18	*PTENP*	Luciferase reporter assay and RNA pull-down assay	Inhibit cell proliferation and EMT and induce cell apoptosis in cervical cancer cells.	[15]
19	*T-UCRs*	qPCR array to profile all 481 T-UCRs in pancreatic cancer specimens and pancreatic cancer cell lines	Expression of T-UCRs in both human and mouse PDAC and a similar mechanism of upregulation in PDAC.	[49]
20	*TUC338*	Plasma, treatment, and cell lines, MS2-MBP protein expression and immobilization	The understanding of molecular mechanisms of lncRNAs. Inhibition of PCSK9 activity is an attractive target for treating the spectrum of sepsis and septic shock.	[33,50]

**Table 2.** Methods to identify lncRNA-associated proteins in cancer.

Method	Description	Strengths	Limitations	Applications in Cancer (Examples)	Key References
RNA pull-down	Biotinylated probes hybridize to lncRNA, isolating associated proteins for MS.	Direct isolation, high specificity.	It requires high RNA abundance and has potential for non-specific binding.	Lung: LncRNA BC promotes lung adenocarcinoma by modulating IMPAD1 splicing. Gastric: *NEAT1/miR-17-5p/TGFβR2 axis drives GC* angiogenesis. Colorectal: lncRNA *FENDRR* suppresses colorectal cancer by binding *GSTP1* and promoting FBX8-mediated ubiquitination.	[61]; [51] [62]
ChIRP-MS	Probes hybridize to chromatin-bound lncRNAs, capturing associated proteins.	Identifies chromatin-associated partners; works for low-abundance lncRNAs.	Limited to nuclear lncRNAs; probe design critical.	Breast: *MaTAR25* modulates Tensin1, influencing breast cancer progression. Gastric: m6A-modified *TP53TG1* suppresses gastric cancer progression by modulating *CIP2A* stability. Liver: LncRNA *CRLM1* cooperates with *hnRNPK* to inhibit apoptosis and promote metastasis in colorectal cancer.	[63] [64]
RAP-MS	Identifies key lncRNA-protein interactions that govern RNA stability, localization, and function.	Yields high-confidence, direct RNA-protein interactions via UV crosslinking and stringent purification.	Requires high RNA abundance and may miss transient or weak interactions.	Liver: lncRNA lincNMR modulates nucleotide metabolism via the *YBX1-RRM2* axis in liver cancer.	[65]
HyPR-MS	Enables multiplexed discovery of specific RNA–protein interactomes.	Versatile method for probing in vivo protein interactomes of target RNAs	Multiplexing capacity beyond three targets and applicability to other RNA species (e.g., rRNA and tRNA) remain untested.	Prostate: *HyPR-MS maps MALAT1, NEAT1,* and *NORAD* interactomes in PC3 cells	[66]
TOBAP-MS	Integrates tobramycin affinity purification with quantitative mass spectrometry.	It enables the isolation of native RNP complexes and the identification of RNA-associated proteins and supports both biochemical and structural studies of these complexes.		Liver: In liver cancer, *HULC*—a lncRNA prominently overexpressed in the disease—engages with 140 interacting proteins.	[34]
BioID-MS	Fuses a promiscuous biotin ligase to a target protein to tag nearby proteins for MS analysis.	Captures transient, weak, and insoluble protein interactions in living cells via biotin tagging for high-affinity purification.	Lower sensitivity, slower labeling kinetics, and higher non-specific biotinylation.	*RNA-BioID in HEK293T* and *MCF-7* cells reveals *HOTAIR’*s association with mitoribosomes, suggesting functions beyond (post)transcriptional regulation.	[55]
SILAC-MS	Uses non-radioactive isotopic labeling to quantify protein abundance differences across samples.	Accurate, multiplexed quantitative proteomics via metabolic labeling with broad proteome coverage and reproducibility.	Restricted to cell culture systems (not applicable to primary tissues/biofluids directly) and limited multiplexing capacity (typically 2–3 samples).	LincNMR promotes tumor proliferation via a *YBX1-RRM2-TYMS-TK1* axis in nucleotide metabolism in liver, lung, and breast cancer cell lines. *GLCC1* drives colorectal cancer through oncogenic mechanisms, functions, and clinical relevance.	[55] [66,67]
